# A possible link to uracil DNA glycosylase in the synergistic action of HDAC inhibitors and thymidylate synthase inhibitors

**DOI:** 10.1186/s12967-020-02555-x

**Published:** 2020-10-07

**Authors:** Meredith S. Showler, Brian P. Weiser

**Affiliations:** 1 Biology Department, Wheaton College, Wheaton, IL 60187 USA; 2 Department of Molecular Biology, Rowan University School of Osteopathic Medicine, Stratford, NJ 08084 USA

**Keywords:** Hdaci, Histone deacetylase inhibitor, Thymidylate synthase inhibitor, UNG2, Uracil DNA glycosylase, p53, 5-Fluorouracil

## Abstract

It is well established that thymidylate synthase inhibitors can cause cellular toxicity through uracil DNA glycosylase (UNG2)-dependent pathways. Additionally, thymidylate synthase inhibitors and HDAC inhibitors are known to act synergistically in a variety of cancer types. A recent article from *J. Transl. Med.* links these together by demonstrating widespread depletion of UNG2 levels across a variety of cell lines treated with HDAC inhibitors. Recent findings suggest that UNG2 depletion by HDAC inhibitors would likely be an effective method to sensitize cells to thymidylate synthase inhibitors. This is particularly important for cancer types that are typically resistant to thymidylate synthase inhibitors, such as cells that are deficient in p53 activity.

## Main text

In a recent article from *J. Transl. Med.*, Iveland et al. showed that HDAC inhibitors caused a comprehensive and widespread depletion of Uracil DNA Glycosylase (UNG2) protein levels in a variety of cancer cell lines [1]. This work has implications for cancer treatments that can be affected by UNG2 activity, namely thymidylate synthase (TS) inhibitors, which include 5-fluorouracil, pemetrexed, and raltitrexed. It is well known that TS inhibitors produce toxicity through UNG2-dependent mechanisms in only certain cancer types [2–5]. We propose that Iveland et al. may have uncovered a novel approach to re-sensitize other cancers to TS inhibitors by targeting UNG2-dependent pathways with HDAC inhibitors.

Since the early 2000s, HDAC inhibitors have been approved for clinical use as cancer treatments, and new generations of molecules continue to be developed. These follow the weak HDAC inhibitor valproic acid, which has been used for decades to treat neurological disorders. Classically, HDAC inhibitors are thought to alter gene transcription by modifying the acetylation state of lysine residues on histone tails, thereby altering chromatin structure and transcription factor access. Transcriptional changes presumably alter the levels of critical proteins that affect cell viability or proliferation [1]. Alternatively, HDACs are known to have many non-histone protein substrates whose acetylation state is also enhanced by HDAC inhibitors, and the acetylation of these proteins can affect their function and stability [6]. Thus, uncovering changes to the proteome and protein post-translational modifications after HDAC inhibitor treatments is an important step in understanding their mechanisms of action and refining their clinical utility.

Interestingly, Iveland et al. found in their proteomic work that treating cancer cells with HDAC inhibitors universally reduced protein levels of UNG2, which is the primary enzyme responsible for removing uracil bases from genomic DNA [1, 7]. The comprehensive depletion of UNG2 was observed in eight human cell lines and was consistent with the reduced UNG2 levels that were reported by others examining HDAC inhibitors in select cell types [8–10]. The reduction of UNG2 was impressively rapid after treatment with a chemically diverse set of molecules (SAHA, MS275, valproate, and sodium butyrate). The reason for reduced UNG2 levels is that HDAC inhibitors induce hyperacetylation of UNG2 [1], which can facilitate its interaction with a ubiquitin ligase [10], thereby resulting in UNG2′s degradation by the proteasome.

Cellular UNG2 levels are important for the toxicity of TS inhibitors, which elevate genomic uracil content by depleting dTTP pools in favor of dUTP [2]. Polymerases then utilize dUTP during replication, which puts the stability of the genome at risk [3, 4, 11]. UNG2 initiates base excision repair pathways to remove genomic uracil and restore DNA to its original code. Therefore, UNG2 activity often mitigates toxicity associated with TS inhibitors in the context of genomic uracilation.

However, the role of UNG2 in TS inhibitor treatments has historically been complex. The toxicity of TS inhibitors in some cell types is entirely independent of UNG2 activity [2, 5]. Recent work has provided a basis for this by demonstrating a strong link between TS inhibitors, UNG2 levels, and p53 activity [5]. Functional wild-type p53 activity is typically associated with a sensitive response of cancer cells to TS inhibitors regardless of UNG2 activity [5, 12]. This is because p53 is important for the activation of apoptosis in cells treated with TS inhibitors [5]. In contrast, cells that are deficient in p53 or harbor mutations that compromise p53 activity are generally resistant to TS inhibitors [5, 12]. However, this resistance can be overcome by depleting UNG2, which elevates genomic uracil content and DNA instability, and promotes apoptosis through p53-independent pathways [3–5].

Interestingly, cells that lack wild-type p53 are typically resistant to TS inhibitors, but can be sensitized to their toxicity by co-treatment with HDAC inhibitors [12]. In fact, HDAC inhibitors and TS inhibitors are well known to behave synergistically against a diverse panel of cancer types [9, 12–14]. This has been attributed to the ability of HDAC inhibitors to also reduce TS expression at the gene and protein level [1, 9, 12, 13], which allows lower doses of TS inhibitor to be efficacious. Indeed, Iveland et al. also measured reduced TS protein levels in cells treated with HDAC inhibitors. It is quite notable that HDAC inhibitors can reduce protein levels of both TS and UNG2, which are critical components of a common pathway governing pyrimidine metabolism and DNA repair [1, 2], and that reduction of TS and UNG2 occur through both transcriptional and post-translational mechanisms [1, 9]. We note that HDAC inhibitors can also impact the expression of additional proteins involved in pyrimidine metabolism, such as thymidine phosphorylase, which regulates the efficacy of other fluoropyrimidine anti-metabolites [15].

We propose that the synergistic action of HDAC inhibitors and TS inhibitors in cells lacking p53 activity is facilitated not only by the downregulation of TS, but also by the reduction of UNG2 levels caused by HDAC inhibitors [12]. Indeed, genetic depletion of UNG2 sensitizes p53 deficient cells to TS inhibitors in a manner that resembles the sensitization caused by HDAC inhibitors [5]. In both cases, a p53-independent apoptosis pathway that is not normally triggered by TS inhibitors becomes activated [5, 12].

The newfound ability to disrupt uracil base excision repair with HDAC inhibitors has immense potential for cancer treatment. UNG2 levels have been pharmacologically reduced with HDAC inhibitors in diverse cell lines that include HT29, HeLa, HEK293, Jurkat, SUDHL5, DLD1, HaCaT, A549, HCT116, and HAP1 [1, 9, 10]. We note that several of these cell lines also lack normal p53 activity and are resistant to TS inhibitors unless they are sensitized pharmacologically or genetically (e.g., HT29 cells which harbor mutant p53) [5, 9]. Providing further pharmacologic specificity is the fact that the selective HDAC inhibitor MS275, which targets HDACs 1–3 [16], reduced UNG2 protein to similar levels as non-selective pan-HDAC inhibitors [1]. This presents a clear path for co-targeting specific HDAC proteins and TS in p53 deficient cells, and evaluating toxicity associated with deficient uracil base excision repair.

The experimental work we discussed could lead to more effective clinical treatments for cancer types that show resistance to TS inhibitors. However, several challenges have been presented thus far during early clinical trials using HDAC inhibitor/TS inhibitor combination therapies of SAHA and 5-fluorouracil [17–19]. Most notably, the well-described ability of SAHA to reduce TS expression in cells has not consistently occurred in solid tumors [1, 9, 12, 13], which hinders proposed mechanisms of SAHA/5-fluorouracil synergy [17–19]. In these clinical trials, it was likely that the SAHA concentrations sustained over time in the tumor were insufficient to reduce TS protein levels, which would not arise as an issue during tissue culture experiments [1]. The ability of SAHA to reduce UNG2 levels in solid tumors has also not been examined. The challenges of targeting these proteins in solid tumors may be overcome with different HDAC inhibitors with improved pharmacologic profiles and/or optimized dosing regimens. This should be combined with biomarker technologies that examine p53 status and measure levels of TS and UNG2 before and after drug treatments. Together, these findings should enhance the promising strategy of targeting pyrimidine metabolism pathways in cancers with diverse p53 status and varying sensitivity to TS inhibitors.

## Data Availability

Not applicable.

## Abbreviations

HDAC: Histone deacetylase
TS: Thymidylate synthase
UNG2: Uracil DNA glycosylase
